# Cavity treatment in primary molars and malocclusion: quasi-randomised clinical trial

**DOI:** 10.7717/peerj.8439

**Published:** 2020-02-07

**Authors:** Rafael T. Gomide, Jo E. Frencken, Jorge Faber, Anne Marie Kuijpers-Jagtman, Soraya C. Leal

**Affiliations:** 1 Division of Pediatric Dentistry, Dental School, Universidade de Brasília, Brasília, Distrito Federal, Brazil; 2 Radboud Institute for Health Sciences, Department of Dentistry—Oral Function and Prosthetic Dentistry, Radboud University Medical Center, Nijmegen, Netherlands; 3 Unaffiliated, Brasília, Distrito Federal, Brasil; 4 Faculty of Dentistry, Universitas Indonesia, Jakarta, Indonesia; 5 Department of Orthodontics, University Medical Center Groningen, Groningen, Netherlands; 6 Department of Orthodontics and Dentofacial Orthopedics, University of Bern, Bern, Switzerland; 7 Department of Dentistry, Faculty of Health Sciences, Universidade de Brasília, Brasília, Brazil

**Keywords:** Non-restorative caries control, Cavity treatment, Primary dentition, Malocclusion, Atraumatic restorative treatment, Restorations, Index of orthodontics treatment index

## Abstract

**Background:**

An innovative caries treatment protocol for primary teeth, termed Ultra-Conservative Treatment (UCT), restores small cavities through the Atraumatic Restorative Treatment (ART) protocol and cleans medium to large open cavities with toothbrush and fluoride toothpaste. However, UCT-treated primary molars were found to exfoliate earlier than amalgam (CRT) and ART-restored cavities, which may lead to unacceptable loss of space for normal eruption of permanent successors. The null-hypothesis tested was that there is no difference between the three treatment protocols and the intra-arch distances, and index of orthodontic treatment need (IOTN) after 4 years.

**Methods:**

Dental casts were taken at baseline (T0) and four (T4) years. The space of the premolars (D + E space), arch perimeter, anterior and total arch depth were measured using a morphometric computer programme. The presence and level of malocclusion were assessed according to the IOTN index. Dependent variables were all intra-arch distances and the IOTN while the independent variable was treatment protocol (CRT, ART and UCT). Data were analysed using linear and logistic regression.

**Results:**

The sample consisted of 867 pairs of casts of 272 initial 6–7-year-olds. No difference was observed between the UCT protocol and the two restorative protocols for the intra-arch variables in both maxilla and mandible over the 4 year period. There was no difference between the UCT and the CRT and ART protocols regarding the occurrence of orthodontic treatment need (malocclusion). In conclusion, the UCT treatment protocol does not differ significantly from the traditional amalgam (CRT) and ART restorative protocols with respect to intra-arch distances and malocclusion. The earlier exfoliation of UCT-treated primary molars does not lead to a worsening of the eruption pattern of permanent successors.

## Introduction

Cavitated dentine carious lesions are very prevalent in primary and permanent dentitions (Marcenes et al., 2013). The conventional restorative protocol (CRT), which uses rotary equipment, has been unable to cure dental caries and to treat its consequences in most cases. More accessible restorative treatment protocols have been developed, such as the Atraumatic Restorative Treatment (ART) and the Ultra-Conservative Treatment (UCT). The UCT protocol, in part, is based on the growing evidence that the caries process in a cavity can be stopped by removing the biofilm from within it regularly with toothbrush and fluoride toothpaste (Kidd, 2012). This procedure is possible in cavities, both occlusal and approximal, that are accessible to a toothbrush and in those that can be enlarged to make access possible (Lo, Schwarz & Wong, 1998). Small tooth cavities are treated with ART within the UCT protocol (Mijan et al., 2014).

A clinical trial that investigated the cumulative survival percentage of primary molars treated through the CRT, ART and UCT protocols did not show a difference over a period of 3.5 years (Mijan et al., 2014). This finding implies that cavities in molars left open but being cleaned in occlusal and approximal surfaces performed as well as comparable cavities restored through either CRT or ART. The study also showed UCT-treated secondary primary molars to exfoliate earlier than CRT- and ART-treated secondary primary molars at year 3 (Mijan et al., 2015). The earlier exfoliation could lead to a larger intra-arch space loss than for the CRT- and ART-treated primary dentition.

It has been suggested that the presence of cavitated dentine carious lesions in primary teeth is associated with malocclusion (Gábris, Márton & Madlénaa, 2006; Mtaya, Brudvik & Astrøm, 2009; Nalcaci et al., 2012) and that severely destroyed primary molars are associated with a reduced space for premolar eruption (Northway & Wainright, 1980). Considering these outcomes, it is reasonable to assume that, although having shown a high percentage of natural exfoliation that was no different from the two restorative treatment protocols, the UCT protocol could eventually lead to malocclusion in the permanent dentition, which would not be acceptable. As the UCT protocol is fairly new, no study has addressed the relationship between this protocol and intra-arch parameters, and occurrence of malocclusion in comparison with cavitated teeth treated restoratively over a long period.

The objective of the study reported on here was to investigate the impact of the CRT, ART and UCT treatment protocols in primary teeth on intra-arch distances and malocclusion over a period of 4 years. The null-hypothesis tested was that there is no difference between the three treatment protocols in intra-arch distances and orthodontic treatment need over 4 years.

## Materials and Methods

### Study design

This study was approved by the Research Ethics Committee of the University of Brasília Medical School (Ref. Nr. 081/2008) and was registered at the Netherlands Trial Registration Centre (Ref. Number 1699). The study design and restorative treatment methods applied in the present investigation have been described in detail elsewhere (De Amorim et al., 2014). A brief description is presented below.

The investigation was a quasi-randomised controlled clinical trial and used a parallel group study design. The subjects were nested in an oral health epidemiological survey of six- and seven-year-old children attending six public primary schools in a socially deprived suburban area (Paranoá) of Brasília, Brazil, from April to May 2009 (De Amorim et al., 2014). Only healthy children having at least two cavitated dentine carious lesions in primary molars without pain and pulp involvement, and whose parents/guardians signed informed consent forms that explained the voluntary nature of participation and the content of the trial were considered eligible for inclusion in the study.

The investigation assessed the exfoliation pattern and survival percentages of primary molars treated according to three treatment protocols: (1) conventional restorative treatment using amalgam (CRT); (2) ART using high-viscosity glass-ionomer; and (3) UCT (Mijan et al., 2014). The UCT protocol consisted of restoring small dentine cavities using the ART method and brushing medium and large cavities biofilm-free using toothbrush and fluoride-containing toothpaste, under the supervision of an assistant during schooldays. Treatment was performed by three paedodontists at the school compound. The interventions were evaluated annually by two independent and trained evaluators.

### Production of study model

Immediately after completion of the restorative treatment, an impression of the upper and lower arch and a wax bite were taken using full autoclavable mouth trays (Morelli^®^, Sorocaba, Brazil) and alginate (Avagel^®^, Dentsply, Petrópolis, Brazil). The impressions were poured in plaster (Asfer^®^, São Caetano, Brazil) within 1 h. These procedures were repeated after 2 (T2), 3 (T3) and 4 years (T4).

Occlusal photographs were taken from the casts with an SLR camera (D40; Nikon, Japan) equipped with a 105 mm Sigma Macro zoom lens (Model EX DG Macro; Sigma, Ronkonkoma, NY, USA). A copy stand with a clear glass top was built so that the camera lens faced up and its long axis remained perpendicular to the glass. Photographs were taken with the casts facing down, with the occlusal plane positioned over the glass. A ruler was placed beside each model and framed in the photograph for calibrating the morphometric programme. All photographs were taken with standardised light and focal distance.

The intra-arch variables, contact point displacement, and tooth impaction were calculated using a morphometric programme (Digimizer v. 4.2; MedCalc Software, Ostend, Belgium) in the upper and lower arches. Overbite and overjet were measured using a 0.02 mm precision dial calliper (Mitutoyo America, Aurora, IL, USA) with the two casts in maximum intercuspation. All the distances were measured by the first author (RG) according to a fixed schedule: every day between 8 AM and 10 AM for about 6 weeks at the same location.

### Description of orthodontic-related variables

The following intra-arch variables were assessed.

#### D + E space

This is the distance from the most mesial point of the first permanent molar to the most distal point of the primary canine in the upper and lower arches (in millimetres) on both sides, with no distinction between sides. Where the first permanent molar was absent, the most distal point of the second primary molar or premolar was taken. If the primary canine was absent, the most mesial point of the first primary molar or premolar was considered. The distance was disregarded if all teeth were missing.

#### Total arch depth

This is the perpendicular distance in millimetres from the line that connects the most mesial point of the right and left first permanent molars to the contact points of the central incisors.

#### Anterior arch depth

This is the shortest distance in millimetres between the line drawn for measuring the inter-canine width and the contact points of the central incisors.

#### Arch perimeter

This is the distance in millimetres of the path that connects the contact point of the right first permanent molar and right second primary molar to the same point on the left side, passing through the contact points on the distal and mesial surfaces of the central incisors and canines.

### Index of orthodontic treatment need

The schoolchildren’s final casts (T4) were graded according to the Dental Health Component of the IOTN index (grade 1–5) and categorised into three treatment groups: no (grades 1 and 2), moderate/borderline (grade 3) and need for orthodontic treatment (grades 4 and 5) by the first author (RG) (Richmond et al., 1992). Treatment was considered necessary when one of the following dental components was presented: unerupted tooth with available space equal to or less than 4 mm; partially erupted tooth; tooth tipped and impacted against adjacent tooth; overjet of greater than 6 mm; anterior or posterior cross bite; contact point displacement or open bite (anterior or lateral) of greater than 4 mm; or overbite with gingival or palatal trauma. Moderate treatment was considered when an overjet was greater than 3.5 mm but less than or equal to 6 mm; contact point displacement or open bite (anterior or lateral) was greater than two but less than or equal to 4 mm; and there was a deep overbite with gingival or palatal contact but no trauma. If the pair of casts did not have one of the dental components described above, it was categorised as ‘no treatment needed’. The IOTN index was related to the following variables.

#### Contact point displacement

Measured between anatomical contact points when the teeth deviate from the line of the arch (Richmond et al., 1992). The largest contact point displacement in both arches was recorded. Displacements between contact points of rotated teeth were not recorded (Richmond et al., 1992).

#### Overbite

This is the vertical distance from the upper central incisor to the incisal edge of the lower central incisor, measured in mm.

#### Overjet

This is the horizontal distance from the buccal aspect of the lower central incisor to the labial surface of the upper central incisor, measured in mm.

#### Tooth impaction

Space measured between two teeth on either side of an unerupted tooth. A distance equal to or shorter than 4 mm was recorded and the unerupted tooth was considered impacted (Richmond et al., 1992).

### Reliability of data measurements

Intra-examiner consistency for measuring the orthodontic-related variables was calculated on 10% of the sample using paired sample correlation. The intra-arch measurements showed a high level of intra-examiner consistency, with correlation coefficients ranging from 0.95 for the lower anterior arch depth to 0.99 for the upper arch perimeter. The calculated weighted κ-coefficient for the intra-examiner consistency test of assessing IOTN-related variables was 0.87, indicating a substantial agreement.

### Statistical analyses

Sample size had been calculated for the cluster-randomised controlled clinical trial and aimed to evaluate the survival rate of primary molars using three restorative treatment protocols (Mijan et al., 2014). In brief, sample size was set at 88 individuals per group (α = 0.05; 1−β = 0.8), including a 10% correction for dependency on treatments within a child, and an 8% estimated annual loss of children (Mijan et al., 2014).

Descriptive statistics of the total arch depth, anterior arch depth and arch perimeter were calculated at baseline (T0), increments (T4–T0) and 4 years (T4). Missing data from the 4 year follow-up were included through the multiple imputation method, using the predictive mean matching method. Dental variables collected but not evaluated in this study were used as auxiliary variables to increase the quality of the imputation. All analyses presented are pooled analyses in the 100-fold imputed data.

Dependent variables were all intra-arch distances and the IOTN while the independent variable was treatment protocol (CRT, ART and UCT). The relationship of the protocols with the intra-arch variables at baseline and between baseline and 4-year evaluation point (T0–T4) were calculated using linear regression while the IOTN and the three treatment protocols were compared using logistic regression. In all regressions analyses the experimental groups were indicated by dummy codes, using the UCT group as reference. The lost-to-follow-up test used the *t*-test for equality of means. The alpha level was set at 5% and all statistics were performed with SPSS and R-statistical software package.

## Results

From the original 302 schoolchildren included in the clinical trial, five were excluded because they had a tooth anomaly (supernumerary or missing lateral primary incisor) and 25 children from the CRT and ART protocols were diagnosed with pulp infection and were not treated restoratively. Five baseline pairs of casts did not match the respective children and a box containing 32 pairs of baseline casts from children treated according to the UCT protocol was lost in transit. All those 37 unavailable pairs of dental casts at baseline had at least one pair of casts at a subsequent year and 19 of them had a pair of casts available at the three subsequent evaluation points (T2, T3 and T4). A total number of 867 pairs of casts were assessed from 272 children. Lost-to-follow-up analyses revealed that the lost-to-follow-up group had slightly lower values for the three parameters measured at T0, however, the differences between the cases that remained in the study and the cases that dropped out were statistically and clinically insignificant. The differences between the two groups were 1.1% for the anterior arch depth, 0.7% for the total arch depth, and 1.0% for the arch perimeter in the maxilla and 3.0% (anterior arch depth), 1.1% (total arch depth) and 1.2% (arch perimeter) in the mandible.

**Figure 1:**
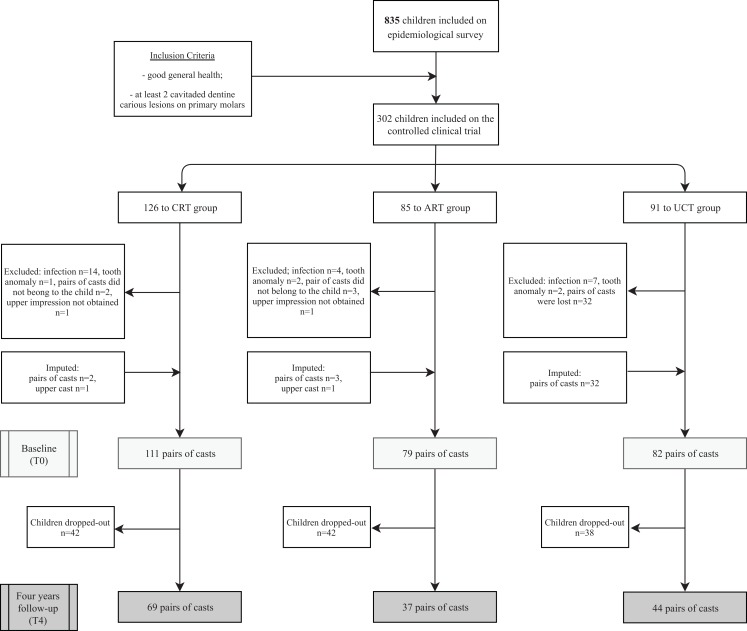
Consolidated Standards of Reporting Trials (CONSORT) flow diagram.

The Consort flow diagram is presented in Fig. 1. Descriptive statistics of the original data at baseline (T0) and at 4-year follow-up (T4), and of the difference between T4 and T0 are presented in Table 1. There was no significant difference between the CRT, ART and UCT treatment protocols regarding age (*p* = 0.053) and gender (*p* = 0.844) at baseline.

**Table 1:** The mean (mm) and Standard Deviation (SD) of intra-arch variables in the maxilla and mandible by treatment (CRT, ART, UCT) at baseline (T0), 4 years (T4) and over 4 years (T4–T0).

Dental arch variables	Treatment protocol	T0		T4		T4–T0		T0		T4		T4–T0	
		Mean ± SD	*N*	Mean ± SD	*N*	Mean ± SD	*N*	Mean ± SD	*N*	Mean ± SD	*N*	Mean ± SD	*N*
		Maxilla						Mandible					
Total arch depth	CRT	26.92 ± 1.91	107	28.17 ± 2.37	69	1.73 ± 1.72	52	24.22 ± 1.71	108	24.20 ± 2.16	69	4.88 ± 1.42	52
	ART	27.00 ± 1.96	75	28.27 ± 2.31	37	1.97 ± 1.75	27	23.95 ± 2.09	76	23.98 ± 1.98	37	5.08 ± 1.82	27
	UCT	26.66 ± 1.77	48	27.87 ± 2.19	44	1.61 ± 1.98	21	24.06 ± 1.47	49	23.69 ± 1.90	44	4.94 ± 1.88	20
Anterior arch depth	CRT	7.48 ± 1.49	105	9.27 ± 1.53	53	1.38 ± 1.34	66	4.34 ± 0.96	105	5.69 ± 1.13	61	4.04 ± 1.42	66
	ART	7.63 ± 1.44	75	9.37 ± 1.47	30	1.34 ± 1.53	33	4.34 ± 0.94	75	5.60 ± 1.02	34	4.14 ± 2.11	34
	UCT	7.36 ± 1.51	48	9.35 ± 1.62	40	1.35 ± 1.57	23	4.23 ± 1.06	46	5.61 ± 1.00	41	4.35 ± 1.72	25
Arch perimeter	CRT	76.43 ± 4.33	107	79.40 ± 5.61	69	2.98 ± 2.24	66	70.06 ± 3.88	108	70.02 ± 4.94	69	9.38 ± 2.23	66
	ART	76.22 ± 4.25	75	79.45 ± 4.85	37	3.10 ± 2.59	33	69.39 ± 4.24	76	70.03 ± 4.42	37	9.29 ± 3.83	34
	UCT	76.09 ± 4.02	48	78.24 ± 4.17	44	2.77 ± 2.50	23	69.48 ± 2.96	49	68.44 ± 3.43	44	10.00 ± 2.69	25

**Note:**

CRT, Conventional Restorative Treatment; ART, Atraumatic Restorative treatment; UCT, Ultra-Conservative Treatment; T0, Baseline; T4, 4-years; *N*, number of quadrants.

### Intra-arch variables assessment

The mean and Standard Deviation in of intra-arch variables in the maxilla and mandible by treatment protocol (CRT, ART, UCT) at baseline (T0), 4 years (T4) and over 4 years (T4–T0) are presented in Table 1. After 4 years, all intra-arch measurements showed the same pattern and they increased over the years in the three treatment protocols. The relationship between dental arch variables and age, gender, mean space at T0 and T4, and treatment protocol for the maxilla and mandible is presented in Tables 2 and 3 respectively. The linear regression analysis showed no difference between the UCT protocol and the two restorative treatment protocols, ART and CRT, in both arches over the 4 years.

**Table 2:** Relationship between dental arch variables and treatment protocol in the maxilla at baseline (T0), 4 years (T4) and over 4 years (T4–T0).

Dental arch variable	Variable	T0			T4			T4–T0		
		Est	95% C.I.	*P*-value	Est	95% C.I.	*P*-value	Est	95% C.I.	*P*-value
Anterior arch depth	(Intercept)	7.08			7.93			1.15		
	Age	0.41	[−0.03…0.86]	0.069	−0.36	[−0.95…0.23]	0.224	−0.77	[−1.45…−0.09]	0.027
	Gender	−0.08	[−0.46…0.29]	0.664	−0.16	[−0.64…0.33]	0.522	−0.07	[−0.63…0.49]	0.813
	ART	0.17	[−0.34…0.68]	0.504	0.10	[−0.47…0.66]	0.740	−0.06	[−0.74…0.62]	0.863
	CRT	0.05	[−0.44…0.53]	0.853	−0.29	[−0.83…0.25]	0.284	−0.32	[−0.96…0.32]	0.319
	DE T0	−0.14	[−0.30…0.02]	0.081	0.31	[0.02…0.60]	0.036	0.48	[0.16…0.79]	0.003
	DE T4				−0.06	[−0.34…0.22]	0.674	−0.11	[−0.43…0.22]	0.512
Total arch depth	(Intercept)	12.85			17.69			5.27		
	Age	0.16	[−0.32…0.64]	0.508	−0.54	[−1.13…0.04]	0.068	−0.70	[−1.30…−0.09]	0.024
	Gender	−0.13	[−0.54…0.27]	0.509	−0.13	[−0.65…0.38]	0.611	0.01	[−0.51…0.54]	0.967
	ART	0.15	[−0.39…0.69]	0.585	−0.11	[−0.78…0.55]	0.735	−0.24	[−0.92…0.44]	0.482
	CRT	0.07	[−0.44…0.59]	0.776	−0.32	[−0.92…0.28]	0.291	−0.37	[−0.98…0.23]	0.225
	DE T0	0.79	[0.62…0.97]	<0.001	0.31	[0.03…0.58]	0.028	−0.46	[−0.73…−0.18]	0.001
	DE T4				0.62	[0.35…0.89]	<0.001	0.55	[0.26…0.85]	<0.001
Arch perimeter	(Intercept)	43.42			51.32			8.11		
	Age	0.25	[−0.73…1.23]	0.612	−0.84	[−1.96…0.29]	0.143	−1.09	[−2.13…−0.05]	0.040
	Gender	−0.84	[−1.67…−0.02]	0.046	−1.20	[−2.18…−0.22]	0.017	−0.35	[−1.28…0.57]	0.448
	ART	−0.36	[−1.48…0.77]	0.532	−0.17	[−1.43…1.09]	0.792	0.20	[−1.01…1.40]	0.747
	CRT	−0.25	[−1.31…0.81]	0.646	−0.21	[−1.35…0.93]	0.720	0.05	[−1.02…1.11]	0.933
	DE T0	1.98	[1.63…2.34]	<0.001	1.04	[0.49…1.59]	<0.001	−0.94	[−1.47…−0.41]	0.001
	DE T4				1.19	[0.66…1.71]	<0.001	1.17	[0.64…1.70]	<0.001

**Note:**

CRT, Conventional Restorative Treatment; ART, Atraumatic Restorative treatment; T0, Baseline; T4, 4 year; DE T0, mean D + E space at baseline; DE T4, mean D + E space after 4 years; Est, estimate; C.I.,Confidence interval.

**Table 3:** Relationship between dental arch variables and treatment protocol in the mandible at baseline (T0), 4 years (T4) and over 4 years (T4–T0).

Dental arch variable	Variable	T0			T4			T4–T0		
		Estimate	95% C.I.	*P*-value	Estimate	95% C.I.	*P*-value	Estimate	95% C.I.	*P*-value
Anterior arch depth	(Intercept)	4.23			6.31			4.97		
	Age	0.29	[0.00…0.58]	0.050	0.00	[−0.40…0.40]	0.999	−0.84	[−1.55…−0.13]	0.020
	Gender	−0.11	[−0.36…0.14]	0.376	−0.16	[−0.48…0.16]	0.327	−0.16	[−0.73…0.42]	0.588
	ART	0.03	[−0.31…0.37]	0.871	−0.01	[−0.42…0.41]	0.969	−0.05	[−0.76…0.66]	0.895
	CRT	0.11	[−0.21…0.43]	0.513	0.08	[−0.30…0.46]	0.688	−0.29	[−0.94…0.36]	0.376
	DE T0	−0.10	[−0.20…0.00]	0.043	0.13	[−0.05…0.31]	0.163	0.15	[−0.13…0.43]	0.289
	DE T4				−0.17	[−0.31…−0.03]	0.022	0.02	[−0.26…0.29]	0.887
Total arch depth	(Intercept)	6.24			12.11			7.00		
	Age	0.19	[−0.20…0.57]	0.340	−0.10	[−0.63…0.43]	0.714	−0.63	[−1.26…0.00]	0.050
	Gender	−0.12	[−0.44…0.21]	0.480	−0.25	[−0.68…0.18]	0.247	0.12	[−0.44…0.67]	0.678
	ART	0.01	[−0.44…0.46]	0.958	−0.13	[−0.69…0.42]	0.638	−0.14	[−0.87…0.59]	0.702
	CRT	0.20	[−0.22…0.63]	0.347	0.25	[−0.26…0.75]	0.334	−0.22	[−0.89…0.45]	0.515
	DE T0	0.94	[0.81…1.07]	<0.001	0.25	[0.02…0.49]	0.037	−0.25	[−0.55…0.04]	0.086
	DE T4				0.53	[0.30…0.76]	<0.001	0.20	[−0.09…0.48]	0.170
Arch perimeter	(Intercept)	29.99			39.45			9.66		
	Age	0.40	[−0.34…1.14]	0.287	−0.41	[−1.41…0.60]	0.425	−0.92	[−2.05…0.20]	0.108
	Gender	−0.87	[−1.49…−0.25]	0.006	−1.00	[−1.82…−0.19]	0.016	−0.11	[−1.12…0.89]	0.823
	ART	0.14	[−0.74…1.02]	0.753	0.38	[−0.70…1.45]	0.491	0.41	[−0.93…1.75]	0.545
	CRT	0.55	[−0.28…1.39]	0.192	0.93	[−0.06…1.92]	0.067	0.37	[−0.83…1.56]	0.546
	DE T0	2.14	[1.88…2.40]	<0.001	0.97	[0.56…1.38]	<0.001	−0.43	[−0.94…0.08]	0.095
	DE T4				1.04	[0.62…1.45]	<0.001	0.45	[−0.05…0.94]	0.075

**Note:**

CRT, Conventional Restorative Treatment; ART, Atraumatic Restorative Treatment; T0, Baseline; T4, 4 years; DE T0, mean D + E space at baseline; DE T4, mean D + E space after 4 years; Est, estimate; C.I., Confidence interval.

### IOTN

The association between the occurrence of malocclusion (IOTN) and age, gender, ART, CRT and mean D + E space at T0 and at T4 in the maxilla and mandible is presented in Table 4. According to the IOTN, orthodontic treatment was needed in 34%, 47% and 36% of the UCT, ART and CRT protocol children respectively. The logistic regression model, with the UCT protocol as reference, showed no difference between the UCT protocol and the two restorative treatment protocols, CRT and ART, regarding the occurrence of orthodontic treatment need.

**Table 4:** Association with UCT as reference between the occurrence of malocclusion (IOTN) and age, gender, ART, CRT and mean D + E space at T0 and at T4 in the maxilla (max) and mandible (mand).

IOTN	OR	95% C.I	*P*-value
(Intercept)	9220.74		
Age	0.50	[0.24…1.05]	0.068
Gender	0.95	[0.50…1.79]	0.872
ART	1.65	[0.70…3.89]	0.247
CRT	1.37	[0.62…3.05]	0.440
Mean DE space T0 max	1.03	[0.69…1.53]	0.890
Mean DE space T4 max	0.67	[0.43…1.03]	0.067
Mean DE space T0 mand	1.10	[0.74…1.64]	0.622
Mean DE space T4 mand	0.89	[0.63…1.25]	0.488

**Note:**

CRT, Conventional Restorative Treatment; ART, Atraumatic Restorative Treatment; UCT, Ultra-Conservative Treatment; OR, Odds Ratio; Gender—boys were scored as 1 and girls as 2.

## Discussion

Restoring a dentine cavity has traditionally been the predominant treatment. In recent years, non-restorative caries control methods for use in primary teeth have been researched on the basis of contemporary cariological principles (Schwendicke et al., 2016). These include Silver Diamine fluoride, ART and the Hall-technique. These methods have shown treatment survival percentages that are comparable with traditional restorations (Santamaría et al., 2018; Tedesco et al., 2017; Mei, Lo & Chu, 2018). The cumulative tooth survival percentage of UCT-treated primary molars was high and not significantly different from those of amalgam (CRT) and ART/high-viscosity glass-ionomer restorations (Mijan et al., 2014). However, whether the UCT protocol would result in a worsening of an existing malocclusion had not been investigated prior to the present investigation.

The present study tested the null-hypothesis that there is no significant difference between the ART, CRT and UCT protocols with respect to intra-arch distances and IOTN after 4 years. The null-hypothesis was accepted. No significant difference concerning these orthodontic variables exists between the UCT and the restorative protocols ART and CRT. The assumption that the earlier eruption of UCT-treated secondary primary molars would cause insufficient space for the premolar to erupt, compared to CRT- and ART-treated primary molars, appears to be unfounded. The ultraconservative treatment (UCT) that removes plaque from accessible tooth cavities in primary molars and restores inaccessible or difficult to access cavities through the ART method is as good as restoring these cavities, with tooth survival as the final end point.

The results are similar to a previous work of Northway & Wainright, 1980. Applying the same methodology of photographs but using a digital software programme of that time, they found out that ‘mild caries cavities’ had no association to tooth migration, while the ‘severed damaged’ primary molars showed earlier exfoliation.

The present study result has important implications for the way dentine cavities in primary teeth can be managed cariologically and how the effect of premature loss of primary molars and tooth migration is considered orthodontically. From a cariological point of view, using UCT in children may increase access to oral care worldwide, lead to cleaner permanent teeth (Hilgert et al., 2017), and reduce dental anxiety and the need for treatment under general anaesthesia. Application of UCT may reduce cost, which is a necessity in terms of healthcare spending and financial resources management (Listl et al., 2015; Vujici, 2018). From an orthodontic point of view, the result of this study indicates that orthodontists can be more tolerant of open cavities in primary teeth, and space maintainers may be less frequently needed. Overall, the UCT treatment protocol appears not to disrupt the development of the occlusion more than do the two restorative treatment protocols.

The strength of the present study includes its relatively long longitudinal aspect, that measurements were taken at the same hours under the same light conditions for a short time period per day using an adequate methodology, and the strong results of the reproducibility tests. The multiple imputation process secured a full set of data that increased the quality of the results. The study also had limitations. The drop-out percentage after 4 years was high, which was mainly because many schoolchildren had moved to other schools by the time of the 4-year evaluation, making it difficult to contact them for a follow-up examination. Furthermore, the schoolchildren had different compositions of mixed-dentition at baseline, which made it difficult to measure tooth migration because of vestibular eruption of incisors. This limitation was circumvented by including the measurement of the D + E space.

## Conclusion

The treatment protocol that consists of cleaning medium- and large-sized occlusal and approximal cavities in primary teeth that are left open with toothbrush and fluoride toothpaste, and restoring small-sized cavities with the ART method does not differ significantly from the traditional amalgam and ART restorative protocols with respect to intra-arch distances and malocclusion over a 4-year period. From an orthodontic point of view, the UCT treatment protocol does not appear to increase the usual space loss that would result in an impairment of the eruption pattern of permanent successors. Future studies should investigate the effect of the UCT protocol in comparison to caries-free teeth with respect to malocclusion.

## Supplemental Information

10.7717/peerj.8439/supp-1 Supplemental Information 1 Data with measurements over 4-years follow-up.

10.7717/peerj.8439/supp-2 Supplemental Information 2 Syntax of the statistics.

10.7717/peerj.8439/supp-3 Supplemental Information 3 CONSORT checklist.

## Additional Information and Declarations

### Data Availability

The following information was supplied regarding data availability: The measurements over the 4 years of follow up and the syntax are available in the Supplemental Files.

### Clinical Trial Registration

The following information was supplied regarding Clinical Trial registration: Netherlands Trial Registration Centre 1699.
